# 1996. Invasive Fungal Infections in Orthotopic Heart Transplant: Incidence and Risk Factors in the Modern Era

**DOI:** 10.1093/ofid/ofad500.123

**Published:** 2023-11-27

**Authors:** Lana Hasan, Kyle D Brizendine

**Affiliations:** Cleveland Clinic, Beachwood, OH; Cleveland Clinic Foundation, Cleveland, OH

## Abstract

**Background:**

Incidence of invasive fungal infections (IFI) in orthotropic heart transplant (OHT) is reported as 3.4–10%. Guidelines from The International Society for Heart and Lung Transplantation recommend considering prophylaxis for invasive molds based on risk factors and local epidemiology. Data defining the risk factors for IFI in OHT patients are lacking. The aim of this study is to describe the epidemiology of IFI in OHT patients and analyze risk factors.

**Methods:**

A retrospective chart review identified all OHT patients who were transplanted at the Cleveland Clinic from 2010-2020. Patients were divided into two groups for comparison: definite or probable IFI within the first year after transplant, and no IFI. We compared the groups to determine independent risk factors for IFI.

**Results:**

We identified 565 OHT patients in the study period; 33 met criteria for IFI with an incidence of 5.8% (95% CI, 4.2–8.1%). **Table 1** shows the characteristics of both patient groups. Among 33 patients with IFI, there were 17 (51%) *Candida*, 9 (27%) *Aspergillus*, 4 (12%) non-Aspergillus mold, and 3 (9%) *Cryptococcus* infections. Median time to IFI (IQR) was 44 days (10–238.5). Specifically, for *Candida* it was 12 days (1-271) and *Aspergillus* was 90 days (8-335). One-year survival was significantly lower in the IFI group (76 vs. 94%; p =< 0.01). Using multivariable logistic regression analyses, as reported in **Table 2**, increased odds of IFI were observed with pre-transplant fungal colonization/infection (OR 19; 95% CI, 2-163) and left ventricular assist device (LVAD) infection (OR 2.3; 95% CI, 0.9-6.0). Post transplant reoperation (OR 4.7; 95% CI, 2-11), and extracorporeal membrane oxygenation (ECMO) (OR 2.6; 95% CI, 1.0-6.7) were also associated with increased odds of IFI.

**Figure:**
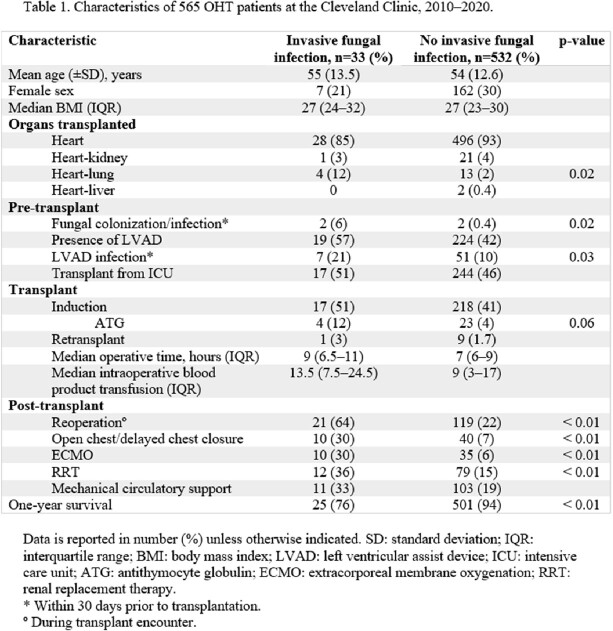

**Figure:**
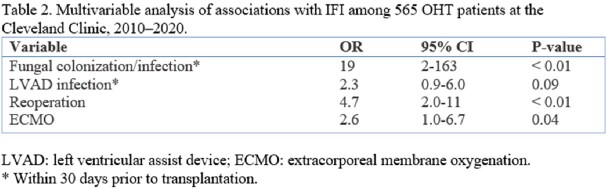

**Conclusion:**

In a large, modern cohort of OHT patients, we observed a significant incidence and increased odds of IFI associated with specific, identifiable risk factors during the pre-transplant and post-transplant periods. These results merit additional study of targeted antifungal prophylaxis with agents offering broad yeast and mold activity in OHT patients with certain risk factors.

**Disclosures:**

**All Authors**: No reported disclosures

